# Long–Term Impairment of Retinal Ganglion Cell Function After Oxygen–Induced Retinopathy

**DOI:** 10.3390/cells14070512

**Published:** 2025-03-29

**Authors:** Adam M. Schmitz, Stephanie M. Bumbaru, Laith S. Fakhouri, Dao-Qi Zhang

**Affiliations:** 1 Eye Research Institute, Oakland University, Rochester, MI 48309, USA; adamschmitz@oakland.edu (A.M.S.); sbumbaru@oakland.edu (S.M.B.); go1935@wayne.edu (L.S.F.); 2 Eye Research Center, Oakland University William Beaumont School of Medicine, Rochester, MI 48309, USA

**Keywords:** cone, bipolar cell, retinal ganglion cell, retinopathy of prematurity, oxygen–induced retinopathy, pattern electroretinogram

## Abstract

Premature infants with retinopathy of prematurity (ROP) have neovascularization of the retina, potentially resulting in low vision and even blindness. Some of these infants still have visual impairment, even if ROP resolves as they age. However, the mechanisms underlying the visual problems post–ROP are poorly understood. Because the pathological neovascularization in ROP infants can be mimicked in a mouse model with oxygen–induced retinopathy (OIR), we recapitulated post–ROP with post–OIR mice a few months after spontaneous regression of retinal neovascularization. Our pattern electroretinogram test demonstrates that post–OIR mice exhibit reduced P1–N2 responses, suggesting the impairment of retinal ganglion cells, the retina’s output neurons. However, immunohistochemistry reveals that the density of retinal ganglion cells remains unchanged in post–OIR mice, indicating that the aforementioned pattern electroretinogram changes are functional. Our data further demonstrate that both light–adapted ex vivo electroretinogram a–waves (cone responses) and in vivo electroretinogram b–waves (ON cone bipolar cell responses) were significantly impaired in post–OIR mice. These results suggest that post–OIR impairment of the retinal cone pathway appears to result in the dysfunction of retinal ganglion cells, contributing to visual problems. A similar cellular mechanism could occur in post–ROP children, which is responsible for their visual impairment.

## 1. Introduction

**Figure 1:**
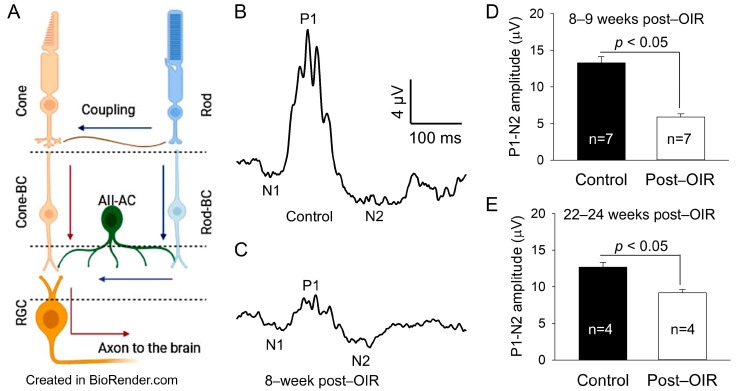
Post–OIR impairment of RGC function in vivo. The neural structure of the mammalian retina is illustrated (**A**), showing that cone signals pass through cone bipolar cells (cone-BC) directly to RGCs, but rod signals reach RGCs indirectly through the rod-cone coupling or via the rod—rod bipolar cell (rod-BC)—AII amacrine cell (AII-AC)—cone-BC pathway. Blue and red arrows indicate the flow of the rod and cone signals, respectively. The function of RGCs was evaluated by PERG in vivo. The PERG used 200 cycles of high–contrast (100%) horizontal gratings with a 1-Hz reversal rate, spatial frequency of 0.0589 cyc/deg, and mean luminance of 100 cd·s/m^2^ as stimuli. Subfigures (**A**,**B**) illustrate typical recordings from an age–matched control mouse and a mouse 8 weeks after OIR, respectively. The P1–N2 amplitude (17 µV) in the control mouse (**B**) was larger than that (5 µV) in the post–OIR mouse (**C**). Data from 8– to 9–week post–OIR mice and aged–matched control mice were pooled and averaged (**D**), suggesting that the P1–N2 amplitude was significantly attenuated in 8–9 weeks post–OIR mice. The attenuation persisted in 22– to 24–week post–OIR mice (**E**).

Visual signals are initiated in rod and cone photoreceptors and transmitted from interneuron bipolar cells to retinal ganglion cells (RGCs) in the retina and then sent from the retina to the brain’s visual centers for image formation (see Figure 1A). The development of retinal neurons and the integrity of visual information processing depend on a rich supply of oxygen and nutrients from ocular blood vessels. Incomplete retinal vascularization in premature infants along with necessary postnatal oxygen therapy can cause abnormal new blood vessel growth, namely neovascularization, due to the overexpression of several growth factors, including vascular endothelial growth factor (VEGF) [1,2]. Retinal neovascularization is the primary feature of retinopathy of prematurity (ROP), an eye problem that can cause low vision and blindness. The visual impairment can remain to some degree, even if ROP resolves spontaneously or after anti–VEGF therapy and photocoagulation treatment. However, the cellular mechanisms underlying post–ROP visual problems are poorly understood.

Several oxygen–induced retinopathy (OIR) animal models have been developed to understand the mechanisms underlying the pathophysiology of ROP [3,4,5,6]. Of these models, mice have been widely used because they are genetically similar to humans and mutant mice share many of the human eye diseases [3,7]. For the OIR mouse model, pups are exposed to high levels of oxygen (e.g., 75% O_2_) for 5 consecutive days from postnatal day 7 (P7) to P12 and then housed in room air (21% O_2_). In this model, oxygen–induced vascular changes occur primarily in the central retina with two disease phases: an initial vaso–obliteration phase with oxygen exposure (P12) and a subsequent hypoxia–induced neovascularization phase (P17). Retinal neovascularization in the OIR mouse model spontaneously regresses and almost fully recovers at P25 [3]. Using OIR mice older than P25 provides an opportunity to understand post–ROP visual problems in premature children.

Visual function changes have been previously evaluated during the retinal neovascularization phase and in post–OIR in mouse and rat models using an in vivo full–field flash electroretinogram (ERG) [8,9,10,11]. During retinal neovascularization or shortly after the regression of retinal neovascularization, both rod– and cone–mediated responses are impaired [8,9,10]. The impaired rod–mediated responses are spontaneously recovered approximately 4 weeks after OIR [11]. However, whether impaired cone–mediated responses are recovered or persist after OIR is unclear. In addition, previous studies have mainly focused on understanding the functional changes of rod and cone photoreceptors and bipolar cells during and shortly after OIR [8,9,11]. However, little is known about how OIR impairs the function of RGCs. RGC function can be evaluated by in vivo pattern ERG (PERG) under light–adapted conditions [12,13]. This noninvasive approach uses equal numbers of reversing black and white checkerboards or gratings as pattern stimuli. Because the overall luminance of the pattern stimuli remains constant, the linear responses for stimulation that contribute to ERG waveforms are thought to be canceled out, leaving only nonlinear responses in the signal. Although the nonlinear pattern responses originate from RGCs, the ON and OFF cone pathways from upstream neurons shape the responses [13,14]. Given the projection of RGCs to visual brain centers, assessing RGC functional changes with PERG and the ON cone pathway would be critical for understanding visual problems in post–ROP children.

The dysfunction of retinal neurons and their neural circuits could be attributed to neuronal loss during and after OIR. In the outer retina, the decreased number of photoreceptors and bipolar cells, shortened outer segments of photoreceptors, and ectopic synapses between photoreceptors and bipolar cells are observed during (P17–P18) and even shortly (e.g., 3 weeks) after OIR, although the results obtained from different investigators varied [10,15]. In the inner retina, dopaminergic amacrine cells are degenerated consistently in both rat and mouse OIR models [16,17,18], whereas the number of RGCs does not change or slightly decreases during or shortly after retinal neovascularization [15,19]. However, whether the changes in cones and RGCs persist long–term after OIR remains unclear.

In this study, we used long–term post–OIR mice in combination with in vivo ERG and PERGs, ex vivo ERG, and immunohistochemistry. We found that RGC function was substantially reduced months after OIR even though its morphology was intact. This reduction is partially attributed to the functional impairment in the ON cone pathway.

## 2. Materials and Methods

### 2.1. Animals

Male and female C57BL/6J mice were obtained for breeding from the Jackson Laboratory (Bar Harbor, ME). In–house bred male and female pups were used for the experiment. The mice were housed in a 12 h light/12 h dark cycle at Oakland University’s Biomedical Research Support Facility. Food and water were given ad libitum. All procedures were performed in accordance with the National Institutes of Health (NIH) guidelines for work with laboratory animals and were approved by the Institutional Animal Care and Use Committee at Oakland University (Protocol #2022–1194).

### 2.2. OIR Mouse Model

Mouse pups were bred and then prepared for OIR. To induce retinopathy, 7–day–old pups and their female dam were placed in a sealed Plexiglas chamber (BioSpherix, New York, NY, USA) for 5 consecutive days. A ProOx P110 oxygen controller (BioSpherix, New York, NY, USA) was connected to the chamber to maintain the oxygen level at 75%. Carbon dioxide levels inside the chamber were managed with Sodasorb (W. R. Grace & Co., Columbia, MD, USA). After 5 days of oxygen treatment, mice were returned to normal room air. OIR pups exhibited retinal vascular changes within 2 weeks [3,16]. The vascular changes were then recovered around P25. Mice older than P25 were referred to as post–OIR mice. In general, mice are considered mature adults between 12 and 24 weeks of age. Therefore, early (12–13 weeks) and late (26–28 weeks) mature OIR mice were assigned for the experiment, which corresponded to 8–9 and 22–24 weeks post–OIR. The same studies were also performed in age–matched control mice. The control mice were maintained in room air from P7 to P12 and kept in the room air afterward.

### 2.3. In Vivo ERG and PERG

Prior to recording, mice were dark–adapted either overnight for in vivo ERGs or 2–3 h for PERGs in a light–sealed chamber. PERGs and ERGs were recorded from anesthetized mice using a Celeris ERG system (Diagnosys, Lowell, MA, USA). Mice were anesthetized with 1.5–2.5% isoflurane (Covetru, Portland, ME, USA) via inhalation. Tropicamide (Akorn, Lake Forest, IL, USA) and phenylephrine (Paragon BioTeck, Portland, OR, USA) were administered to the eyes of an anesthetized mouse for pupil dilations. GenTeal Tears Eye drops (Alcon, Fort Worth, TX, USA) were applied every 5 min to keep the eyes moist.

For ERG recording, a dual–function electrode stimulator was placed onto each eye. The electrode–stimulator controlled by Diagnosys Espion™ software (https://www.diagnosysllc.com/espion-software/, accessed on 1 January 2025) (Diagnosys, Lowell, MA, USA) simultaneously delivered light stimuli and recorded neural activity. To evaluate rod–mediated responses, ERGs were recorded in response to light pulses (white light, 4 ms) with −2 log cd·s/m^2^ (1 log unit = 10 cd·s/m^2^) and −1 log cd·s/m^2^, respectively. These intensities are below the stimulus threshold of cone photoreceptors (0.5 log cd·s/m^2^). Therefore, the recorded ERG a– and b–waves were purely mediated by rod photoreceptors.

To evaluate cone–mediated visual function, 40 cd·s/m^2^ white background illumination was applied for 10 min to saturate the rod response and light–adapt the retina. In the presence of background light, a series of light pulses (white, 4 ms) with intensities ranging from 0.5 to 3.0 log cd·s/m^2^ were then delivered every 10 s. Responses induced by light pulses (a– and b–waves) were recorded at a sample rate of 2 kHz. Light responses recorded from the right and left eyes were analyzed separately (see Section 3). Data from both eyes were averaged for each mouse tested.

For PERG recording, a dual–function PERG electrode (Diagnosys, Lowell, MA, USA) was placed on the right eye. The electrode provided high–contrast (100%) horizontal gratings with a 1-Hz reversal rate, spatial frequency of 0.0589 cyc/deg, and a mean luminance of 100 cd·s/m^2^ to the eye. Two hundred cycles were run, and the data were recorded at a sample rate of 2 kHz. Data from 200 cycles were averaged and presented (see Section 3).

### 2.4. Ex Vivo ERG

Ex vivo ERG recording procedures were identical to those described previously [20]. In brief, retinas were dissected from euthanized mice and placed in a recording chamber perfused with Ames’ medium (US Biological, Salem, MA, USA)_._ To record transretinal ERGs, two Ag/AgCl electrodes were placed in the photoreceptor and RGC sides. Two intensities (−6.29 and −4.84 log photons/cm^2^/s; 0 log unit = 2.20 × 10^16^ photons/cm^2^/s) of full–field 470 nm light pulses (20 ms) were delivered to the retina. Because these intensities are below the stimulus threshold (−2.82 log photons/cm^2^/s) of cone photoreceptors, so light–evoked a– and b–waves were mediated only by rod photoreceptors.

To obtain light–adapted ERGs, light adaption of the retina was achieved with a combination of steady background illumination (to saturate rod responses) and repetitive light pulses (to induce the light–adapted state of the retina). After 5 min light adaptation, ERG a–waves were recorded in response to a series of light intensities while b–waves were blocked with 20 µM L–(+)–2–Amino–4–phosphonobutyric acid (L–AP4, Hello Bio, Princeton, NJ, USA) [21]. The ERG waveforms were amplified using a Grass amplifier (Grass Telefactor, Warwick, RI, USA) and acquired using Clampex 10 software (Molecular Devices, Sunnyvale, CA, USA). The data were analyzed offline using Clampfit 10 software (Molecular Devices, Sunnyvale, CA, USA).

### 2.5. Immunohistochemistry

Mice were euthanized by asphyxiation with CO_2_ followed by cervical dislocation. Immediately after euthanasia, both eyes were enucleated, fixed in 4% paraformaldehyde (Sigma, St. Louis, MO, USA), and dissected. During retinal dissection, two landmarks were used to identify retinal orientation [22]. One was the choroid fissure on the sclera (the white line across the eyeball) that runs from the temporal to the nasal pole. Another was the optic nerve head that is located on the dorsal side, proximal to the choroid fissure. Whole retinas were used for the immunostaining of the short– and middle–wavelength cones (S–cones and M–cones). For immunostaining of an RGC marker RPBMS (RNA binding protein with multiple splicing), whole retinas were treated with 20% sucrose in PBS for 2 h and then moved to 30% sucrose at 4 °C overnight. The following day, retinas were transferred to a −80 °C freezer until frozen. They were then moved to room temperature to thaw. This procedure was repeated three times.

Whole retinas were blocked for 2 h with 1% bovine serum albumin (Fisher Scientific, Hampton, NH) and 0.3% Triton–X 100 (Sigma, St. Louis, MO, USA) in 0.1X PBS (VWR, Radnor, PA, USA). Retinas were incubated with a primary guinea pig anti–RPBMS antibody (1:200; Phosphosolutions, Aurora, CO, USA), a rabbit anti–red/green opsin antibody (1:1000, Millipore Sigma–Aldrich, Burlington, MA, USA), or a rabbit anti–S–opsin antibody (1:1000, Millipore Sigma–Aldrich, Burlington, MA, USA) for a minimum of 48 h. Retinas were then rinsed in 0.1× PBS and incubated for 2 h with a secondary antibody raised in donkey, conjugated to Alexa Fluor–488 or Alexa Fluor–594 (1:500; Life Technologies, Carlsbad, CA, USA). Finally, whole retinas were mounted on slides with Vectashield Hard–Set Mounting Solution (Vector Laboratories, Burlingame, CA, USA).

### 2.6. Imaging and Analysis

Whole–mount retinas were visualized on a ZEISS LSM 900 with Airyscan 2 Confocal Microscope using Zeiss Zen software (https://www.zeiss.com/microscopy/en/products/software/zeiss-zen.html, accessed on 1 January 2025) to take images (Zeiss, Oberkochen, Germany). Images were taken using 40× or 63× magnification. Image analysis and processing were also performed using Zeiss Zen software.

The RGCs were imaged from each of the four quadrants of the retina (superior, inferior, nasal, and temporal) at a distance of 0.7 mm and 2.0 mm from the optic disc. These distances were chosen in order to evaluate the RGC density in the center and peripheral regions, respectively, because vascular changes occur primarily in the central retina of the OIR mouse [3]. Each of the images were then analyzed and manually counted through the NIH ImageJ software (https://imagej.net/nih-image/, accessed on 1 January 2025). The RGC density was calculated by dividing the number of RGCs by the counted area. The densities from four quadrants were averaged to obtain the mean density for one mouse.

In the pigmented mice, S–opsin was predominant in the ventral retina, whereas M–opsin was evenly distributed across the retina [23,24,25]. Therefore, S–opsin–expressing cones were imaged at a distance of 0.5 and 2.0 mm from the optic disc in the ventral region, whereas M–opsin–expressing cones were imaged in the dorsal retina. The methodology for counting and calculating these cones’ density was the same as that of RGCs.

### 2.7. Statistical Analysis

A Student’s *t*–test was used to compare two independent groups. Values are presented as the mean ± SEM in the present study, and *p* < 0.05 was considered statistically significant.

## 3. Results

### 3.1. The P1–N2 Amplitude of the PERG Was Attenuated in Long–Term Post–OIR Mice

OIR mice had retinal neovascularization that peaked around P17. The neovascularization then spontaneously regressed and almost completely recovered around P25 [3,16]. To determine whether RGCs (Figure 1A) undergo dysfunction after OIR, we used OIR mice 8–9 weeks after the regression of retinal neovascularization and age–matched controls. We evaluated the function of RGCs using an in vivo PERG. PERG in mice exhibits negative N1, positive P1, and negative N2 responses to a pattern stimulus [9]. Because the P1 and N2 responses are thought to originate from RGCs, we measured the absolute difference in amplitude between the positive peak of P1 and the negative trough of N2. We referred to it as P1–N2 (µV), a key indicator of RGC function. Figure 1B,C illustrate typical recordings from control and post–OIR mice, respectively. The results show that the P1–N2 amplitude in the post–OIR mouse was smaller than that of the control mouse. We pooled and averaged data from mice 8–9 weeks after OIR and control mice. The mean data show there was an approximately 55% reduction in P1–N2 amplitudes in post–OIR mice (5.90 ± 0.42 µV) compared to control mice (13.28 ± 0.87 µV) (Figure 1D).

To determine whether this reduction persists longer than 8–9 weeks after OIR, we performed similar recordings from OIR mice 22–24 weeks after OIR and age–matched control mice. Compared to 8–9 weeks post–OIR, the reduction in P1–N2 amplitude in 22–24 weeks post–OIR was reduced from 55% (Figure 1D) to 28% (Figure 1E). However, this reduction (28%) still revealed a significant difference between post–OIR (9.18 ± 0.44 µV) and control mice (12.69 ± 0.63 µV) (Figure 1E). Our data demonstrate that the function of RGCs remains impaired relatively long after OIR.

### 3.2. The Density of RGCs Remains Unchanged in Long–Term Post–OIR

**Figure 2:**
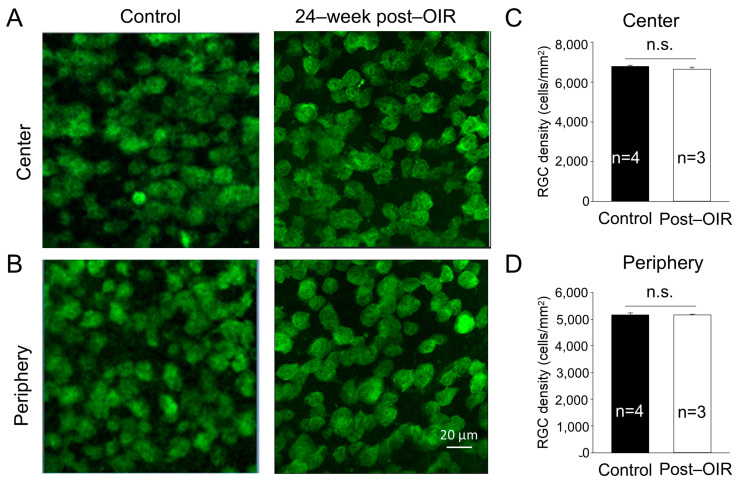
No significant changes of the RGC density in post–OIR mice. RGCs were immuno–stained with an antibody against RPBMS in whole–mount retinas isolated from age–matched mice and mice 24 weeks after OIR. Images were taken from the center (**A**) and peripheral region (**B**) of the retina. The number of RGCs was manually counted and then divided by the counted area to find the RGC density. The average RGC density in the center and periphery was depicted in (**C**,**D**), respectively. n.s., no significance.

One possibility causing the impairment of RGC function is the loss of RGCs. Previous studies have shown that the density of RGCs showed no changes or slight decreases during neovascularization or shortly after OIR [15,19]. To determine whether long–term post–OIR causes the loss of RGCs, we collected retinas from OIR mice 24 weeks after OIR and age–matched control mice. We performed immunohistochemistry with an antibody against RBPMS. We imaged RBPMS–positive cells from the center and periphery of the retina (see Section 2). Individual images showed no apparent changes in the number of RGCs in both the center and peripheral regions of the OIR retina 24 weeks after OIR (Figure 2A,B, right panels) compared to that in the age–matched control (Figure 2A,B, left panels). We then manually counted the number of cells and divided that number by the cell counting area to determine the cell density. We found that the densities of RGCs remained unchanged in the center (Figure 2C) and peripheral (Figure 2D) regions of the retina between post–OIR and control mice. No significant RGC loss in post–OIR mice suggests that functional changes of RGCs are attributed to other causes, such as dysfunctions of upstream neurons.

### 3.3. Light–Adapted ERG b–Waves Are Attenuated in Long–Term Post–OIR

**Figure 3:**
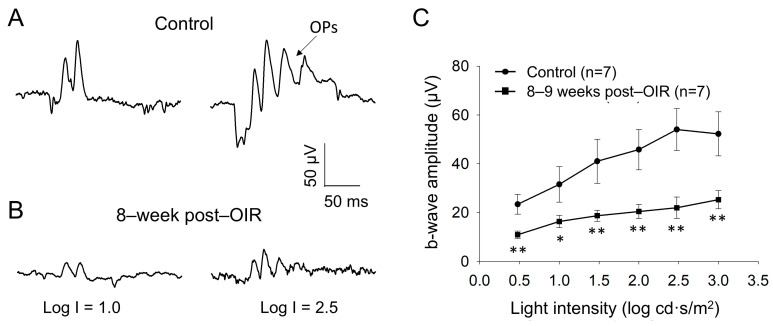
Reduced b–waves of in vivo light–adapted ERGs in post–OIR mice. Light–adapted full–field ERG a– and b–waves were recorded from age–matched controls (**A**) and 8–week post–OIR mice (**B**). Protocol details and ERG waveforms (a– and b–waves and OPs) are described in the main text. The intensity-response curve of b–waves was constructed by plotting its amplitude as a function of the stimulus intensity (**C**). * *p* < 0.05; **p < 0.01. 1 log unit = 10 cd·s/m^2^.

Although PERG responses originate from RGCs, they are shaped by input from upstream neurons (see details in Section 4) [14]. PERGs are routinely assessed under light–adapted conditions, which leads to the hypothesis that the cone pathway formed by cones and cone bipolar cells is impaired during the long–term post–OIR. To test this hypothesis, we examined light–adapted ERG from mice 8–9 weeks after OIR and age–matched controls in vivo. We delivered background illumination with an intensity saturating rod response to an eye for 10 min to light–adapt the retina. While the background light was continually delivered, we then applied a series of light pulses with intensities from 0.5 log cd·s/m^2^ to 3.0 log cd·s/m^2^ to the eye to evoke light–adapted ERGs. Figure 3A illustrates typical traces of light–adapted ERG at low (1.0 log cd·s/m^2^) and high (2.5 log cd·s/m^2^) intensities from a control mouse. In addition to a– and b–waves, there were oscillatory potentials (OPs) on the rising phase of the b–wave. Compared to the control mouse (Figure 3A), light–induced ERGs evoked by both intensities in a post–OIR mouse were attenuated (Figure 3B).

Because light–adapted a–waves may be predominantly contributed by post–receptoral neurons from the OFF pathway, rather than cone photoreceptors [26,27,28], we did not further analyze them. In addition, OPs largely merged into the b–wave, and they varied from animal to animal. We therefore measured the amplitude of the b–wave from the trough of a–waves to the peak of OPs to keep the consistency of the measurement of b–wave amplitude. With this measurement, there was a systemic error in that the estimated b–wave amplitude was higher than the actual value. Despite the higher values, the key findings are still expected to be valid because OPs arise from neural circuits in the inner retina, especially the interactions between bipolar cells, amacrine cells, and RGCs [29]. After measurement, we plotted the amplitude of b–waves over the intensity and found that the amplitudes of b–waves were significantly reduced in every intensity of light pulses tested (Figure 3C). The results suggest that the ON cone bipolar cell function remains impaired during long–term post–OIR, which could contribute to the dysfunction of RGCs evaluated by PERGs.

### 3.4. Ex Vivo ERG a–Waves Are Decreased Under Long–Term Post–OIR

**Figure 4:**
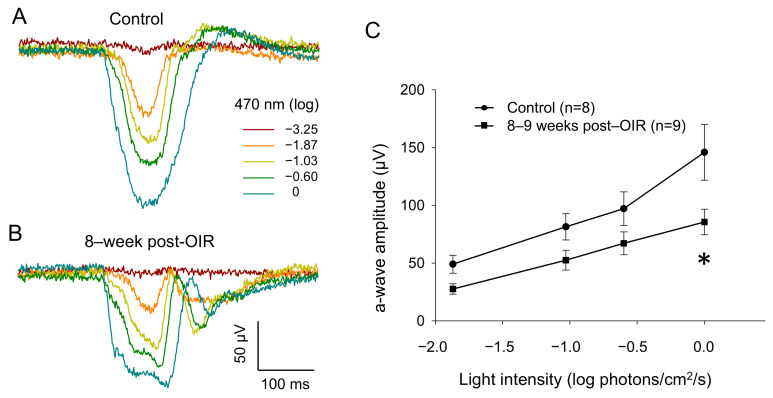
Reduced a–waves of ex vivo light–adapted ERGs in post–OIR mice. Light–adapted a–waves were recorded from isolated retinas of age–control mice (**A**) and 8–week post–OIR mice (**B**), whereas b–waves were pharmacologically eliminated. The procedures of retina preparation and recording protocols are described in the main text. The peak amplitudes of a–waves were plotted as a function of the stimulus intensity to construct an intensity response curve (**C**). * *p* < 0.05, 0 log unit: 2.20 × 10^16^ photons/cm^2^/s.

To determine whether cone function remains impaired under long–term post–OIR conditions, we switched our ERG recording from in vivo animals to ex vivo retina preparation [20]. Using this preparation, we were able to isolate an a–wave by pharmacologically blocking the b– wave with L–AP4, an agonist that selectively acts on group III metabotropic glutamate receptors on ON–bipolar cells [21]. As described in the Section 2, we delivered background light (470 nm, −2.82 log photons/cm^2^/s) to saturate rod responses. Simultaneously, we delivered 1–Hz flashing lights (470 nm, 20 ms, 0 log photons/cm^2^/s) for 5 min so that we could light–adapt the retina in order to obtain fully light–adapted cone responses. Figure 4A,B illustrate original traces of a–waves from a control retina and an 8–week post–OIR retina in response to a series of light intensities from −3.25 to 0 log photons/cm^2^/s (470 nm, 20 ms) in the presence of background light. The data show that the a–wave evoked with each intensity in the post–OIR retina was smaller in amplitude than that in the control retina. We further measured the peak amplitude of the a–wave and pooled the data collected from 8– to 9–week post–OIR retinas or from age–matched control retinas. We constructed an intensity response curve by plotting the mean peak amplitude of the a–wave at each intensity along with light intensities (Figure 4C). The curves show that the peak amplitude at each intensity in post–OIR mice was lower than that in control retinas. However, only the change in the highest intensity was statistically significant. Our data suggest that reduced cone function under post–OIR may partially contribute to the impairment of light–adapted ERG b–waves and P1–N2 of PERG.

### 3.5. The Density of M–Opsin–Expressing Cones Remains Unchanged in Long–Term Post–OIR

**Figure 5:**
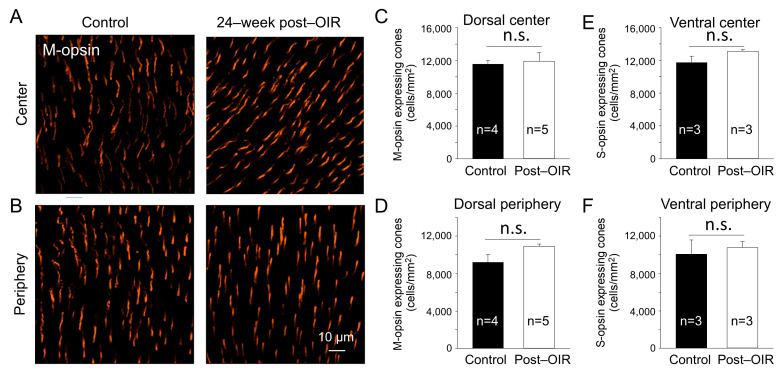
No significant changes of the cone density in post–OIR mice. S–opsin–expressing and M–opsin–expressing cones were immunostained in whole–mount retinas isolated from age–matched mice and mice 24 weeks after OIR. Images were taken for M–opsin–expressing cones from the center (**A**) and peripheral region (**B**) of the dorsal retina. The number of M–opsin–expressing cones was manually counted and then divided by the counted area to find the density. The average M–opsin–expressing cone density for the center and periphery was depicted in (**C**,**D**), respectively. Similar procedures were used for S–opsin–expressing cone imaging from the ventral retina where S–opsin is predominantly expressed. Subfigures (**E**,**F**) show the average S–cone density for the center and periphery, respectively. n.s, no significance.

In the mouse retina, S–cones have peak responses around 360 nm in the ultraviolet (UV) spectrum, whereas the M–cones peak around 508 nm in the visible light spectrum [30]. To determine whether the reduction in cone responses to 470 nm visible light in post–OIR was due to the loss of M–opsin–expressing cones, we performed immunohistochemistry with an antibody against M–opsin using 24–week post–OIR and age–matched control mice. Images were taken from the center (Figure 5A) and peripheral (Figure 5B) regions of the dorsal retina. Compared to the controls (Figure 5A,B, left panels), there were no notable changes in the number of cones in post–OIR mice (Figure 5A,B, right panels). Average data show no significant changes in the number of M–opsin–expressing cones at the retina’s center (Figure 5C) and periphery (Figure 5D).

S–cones appear to be more susceptible to specific insults than M–cones [31]. To ensure that the cones survived in post–OIR mice, we also immunostained S–opsin–expressing cones in 24–week post–OIR and age–matched control mice. The densities of S–opsin–expressing cones in the center and periphery of the ventral retina in post–OIR mice showed no significant changes compared to control mice (Figure 5E,F). Together, the data suggest that cone photoreceptors are resilient during post–OIR, which cannot explain the decreased a–waves in ex vivo ERGs.

### 3.6. Long–Term Post–OIR Alters Scotopic b–Waves but Not a–Waves In Vivo and Ex Vivo

**Figure 6:**
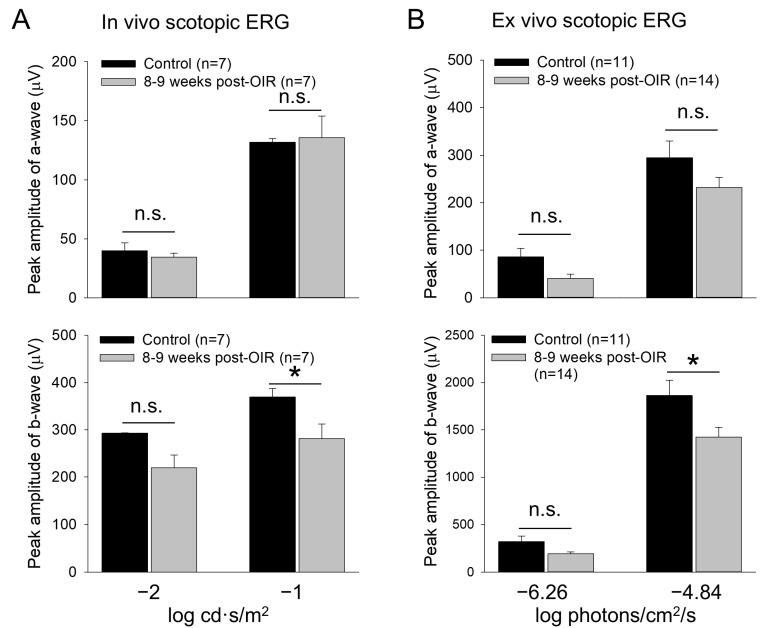
Reduced b–waves of in vivo and ex vivo scotopic ERGs in post–OIR mice. (**A**). In vivo dark–adapted full–field ERG a– and b–waves were recorded from 8– to 9–week post–OIR and age–matched control mice in response to 4 ms white light pulses with −1 or −2 log cd·s/m^2^ (1 log unit = 10 cd·s/m^2^), only stimulating rod photoreceptors. The peak amplitude of a–waves remained unchanged at both intensities (top panel). Significant changes in b–waves were observed at high intensity but not at low intensity (bottom panel). (**B**). Ex vivo dark–adapted ERGs showed similar results as in vivo dark–adapted ERGs. Top panel, a–waves; bottom panel, b–waves. The 470 nm light pulses with a 20 ms duration were applied at −6.26 or −4.84 log photons/cm^2^/s. Both intensities only stimulated rod photoreceptors. *, *p* < 0.05; n.s., no significance.

The lack of apparent changes in the cone density suggests that the reduction in cone responses could be due to a disruption of the cone neural network such as rod-cone electrical coupling (Figure 1A). Through the coupling, rods disinhibit cones upon background illumination, causing network light adaption of cones [32]. Therefore, any defects in rods could result in cone dysfunction under post–OIR. To test this possibility, we revisited the previously reported recovery of rod function under post–OIR conditions [11]. We performed in vivo and ex vivo dark–adapted ERG recordings from 8– to 9–week post–OIR mice and age–matched controls. As described in the method, we only tested light intensities that were below the threshold intensity of cone photoreceptors. Therefore, the light–evoked a– and b–waves obtained were mediated purely by rods. As shown in Figure 6A, in vivo ERG showed that the amplitude of a–waves in post–OIR mice remained unchanged compared to control mice (top panel). However, the amplitude of b–waves was decreased by 23.8% from 369.3 ± 18.4 µV in the control (n = 7) to 281.4 ± 30.5 µV in post–OIR (n = 7, *p* < 0.05) at the −1 log cd·s/m^2^ intensity (Figure 6A, bottom panel).

Similar results were obtained from ex vivo ERGs (Figure 6B). The amplitudes of a–waves at low (−6.26 log photons/cm^2^/s) and high (−4.84 log photons/cm^2^/s) intensities tended to decrease but the decreases were not significant (Figure 6B, top panel). The change in the amplitude of b–waves was also not significantly changed at the low intensity. However, the peak amplitude of b–waves was decreased by 23.7% from 1863 ± 161 µV in control mice (n = 11) to 1421 ± 105 µV in post–OIR mice (n = 14, *p* < 0.05) at the high intensity (Figure 6B, bottom panel). Taken together, our in vivo and ex vivo data collected at 8– to 9–week post–OIR mice do not entirely agree with the previous finding that rod function is fully recovered 4 weeks after OIR [11]. The persistent impairment of rod–mediated responses of rod bipolar cells could contribute to RGC dysfunction via the cone signaling pathway under the light–adapted state of the retina.

## 4. Discussion

The OIR mouse model has been widely used for ischemic retinopathy, mimicking retinal neovascularization in ROP. With this model, visual function changes have been evaluated mostly during retinal neovascularization and shortly after the regression of retinal neovascularization [10,15]. Unlike the earlier work, the present study mainly focused on assessing visual impairment during the long–term post–OIR. Under this condition, we found that cone–mediated visual signals remain impaired in cones, ON cone bipolar cells, and RGCs. The results provide insights into the cellular mechanisms underlying visual impairment under relatively long–term post–OIR conditions.

### 4.1. Evaluation of Visual Function with Multiple ERG Approaches

The retinal function can be evaluated in vivo directly from the eye of a living organism or ex vivo from isolated retinas. However, the application of these two approaches can be different. The former is widely used for diagnosing retinal diseases in patients and studying changes in visual function in experimental animals [33,34]. In contrast, the latter is employed to investigate the function of retinal neuron classes, including rods, cones, and bipolar cells, and evaluate potential therapeutic drugs for retinal diseases [20,35,36,37]. The in vivo ERG has been previously used to assess visual dysfunction in OIR animal models [10,15]. One of the limitations of in vivo ERGs is that they cannot determine cone responses because light–adapted a–waves are predominantly mediated by the activity of the post–receptoral OFF pathway [26,27,28]. To overcome this limitation, the present study took advantage of ex vivo ERG recording in combination with pharmacology to evaluate cone photo–responses in post–OIR, providing a tool for visual function assessment in OIR animal models. In addition, the a– and b–waves of the ERGs only reflect the visual function in the outer retina, leaving the effect of OIR on RGCs unknown. This study has filled the research gap by employing PERG recordings in order to evaluate the changes in RGC function in post–OIR retinas. Because RGCs are the retina’s output neurons, the changes in their activity provide a more accurate reflection of how OIR impacts visual behavior and perception.

### 4.2. Functional Impairment of RGCs in Post–OIR

A previous study has shown that the loss of RGCs induced by optic nerve crush can eliminate the PERG without changing light–induced a– and b–waves from the outer retina, suggesting that RGCs are the origin of PERGs [14]. Such a study indicates that the reduced P1–N2 responses of PERGs in post–OIR mice occur in RGCs. The RGC dysfunction could be attributed to the loss of RGC and/or their dendritic alteration. The former is unlikely because the density of RGCs did not change significantly post–OIR. A future study is needed to determine whether RGCs undergo dendritic alterations that potentially cause an impairment in RGC function.

In addition, the input from ON and OFF bipolar cells can regulate RGC activity associated with generating PERGs [13,14]. Indeed, we found the activity of ON cone bipolar cells, as evidenced by in vivo light–adapted ERG, was suppressed under post–OIR conditions. The suppression of ON cone bipolar cell activity could significantly cause the decreased P1–N2 responses of PERG in post–OIR retinas. Moreover, the blockade of Na^+^–dependent spiking activity has been shown to substantially reduce the amplitude of P1 and N2 responses, suggesting that spiking activity, mostly on RGCs, plays a vital role in generating PERGs [13,14]. The decreased b–waves of light–adapted ERGs suggest that ON cone bipolar cell–driven spiking activity on RGCs may be attenuated in post–OIR retinas. This attenuation can cause the reduction in P1 responses. It is worth noting that our data cannot rule out the possibility that the intrinsic spiking activity of RGCs is altered under post–OIR conditions, resulting in a change in PERG. This possibility can be tested with single RGC recordings in a future study.

Furthermore, we did notice that the PERG reduction was progressively recovered from 8–9 weeks to 22–24 weeks post–OIR. Does this trend suggest that RGC function is able to be fully recovered at later time points? It is apparent that older post–OIR mice need to be tested to address the question. If the experiment were performed, we speculate that this is unlikely as mice at 7 months are already considered to be in the late adult stage.

### 4.3. The Contribution of the Cone Pathway to the Impairment of RGCs

The approximate 55% decrease in P1–N2 amplitude of PERGs could be interpreted as a similar degree of reduction in light–adapted ERG b–waves under post–OIR conditions. The reduction in the b–wave amplitude could be caused by the loss of cone bipolar cells, less input from cones, or the impairment of light–adaptation of the bipolar cell neural network. Because of the lack of pan–antibody staining of cone bipolar cells, we were unable to determine whether the loss of cone bipolar cells occurred in post–OIR mice. However, we found that the photo–responses of cone photoreceptors were decreased in post–OIR mice. This decrease appears to be functional as the densities of S–opsin–expressing and M–opsin–expressing cones were close between control and post–OIR mice. The data suggest that less functional input from cone photoreceptors could contribute to the decreased activity of cone bipolar cells. It is worth noting that cone responses were evaluated using ex vivo ERG and that there was only a 20–30% decrease in the amplitude of the responses. This percentage of the decrease in ex vivo cone responses was less than that of in vivo bipolar cell responses. Although we cannot rule out that this difference was caused by different stimuli, recording parameters, or the preparation environment between in vivo and ex vivo ERG recording, the data suggest that the reduced cone photo–responses partially contribute to the loss of ON cone bipolar cells. The remaining suppression of ON cone bipolar cells could be due to the alteration of the cone bipolar cell synapse or the decrease in the network adaptation of cone bipolar cells. Dopamine is a key neuromodulator of retinal network adaptation [38]. The persistent loss of dopaminergic amacrine cells and dopamine deficiency in post–OIR retinas [16,17] could impair light–adapted responses in cone bipolar cells, which needs further investigation.

### 4.4. Possible Contribution of the Rod Pathway to Dysfunction of RGCs

Our in vivo and ex vivo ERG data agree with the previous work demonstrating that scotopic a–waves are recovered post–OIR [11], suggesting rod photoreceptors are morphologically intact. This further supports the fact that no significant loss of S–opsin–expressing and M–opsin–expressing cones was observed post–OIR, as the cone loss often occurs after the loss of rods during retinal degeneration [39]. Inconsistent with the previously reported full recovery of scotopic b–waves [11], we found they were diminished to some degree, suggesting there could be minor damages in rod function. If this is the case, the strength of rod disinhibition of cone light adaptation through rod-cone coupling (Figure 1A) could be reduced [32]. This reduction could contribute to the diminished responses of cones observed under post–OIR conditions (Figure 4), which in turn affects RGC function. Moreover, the decreased scotopic and photopic b–waves suggest that both rod bipolar and cone bipolar cells are still vulnerable long–term after OIR. Future investigations are needed to systemically assess the loss of rod bipolar cells and cone bipolar cells, and determine the possible alterations in their synaptic connections with photoreceptors and RGCs.

## 5. Conclusions

This study, for the first time, has revealed the persistent impairment of RGCs under long–term post–OIR conditions, possibly caused by the reduction in cone–mediated signal transmission from the outer to the inner retina. These findings provide a novel mechanistic insight into visual impairment in an experimental ROP model. Moreover, this mechanism may offer a valuable explanation for lifelong visual deficits observed in children with a history of ROP [1,2].

## Data Availability

The data presented in this study are available on request from the corresponding author.

## Abbreviations

The following abbreviations are used in this manuscript:

ROP	Retinopathy of prematurity
OIR	Oxygen–induced retinopathy
ERG	Electroretinogram
PERG	Pattern electroretinogram
RGC	Retinal ganglion cell
VEGF	Vascular endothelial growth factor
L–AP4	L–(+)–2–Amino–4–phosphonobutyric acid
RPBMS	RNA binding protein with multiple splicing
OPs	Oscillatory potentials
